# Nicotinamide Phosphoribosyltransferase as a Key Molecule of the Aging/Senescence Process

**DOI:** 10.3390/ijms22073709

**Published:** 2021-04-02

**Authors:** Fiqri D. Khaidizar, Yasumasa Bessho, Yasukazu Nakahata

**Affiliations:** 1 Centre for Research in Biotechnology for Agriculture (CEBAR), University of Malaya, Kuala Lumpur 50603, Malaysia; 2 Laboratory of Gene Regulation Research, Division of Biological Science, Graduate School of Science and Technology, Nara Institute of Science and Technology (NAIST), Ikoma 630-0101, Japan; ybessho@bs.naist.jp; 3 Department of Neurobiology & Behavior, Nagasaki University Graduate School of Biomedical Sciences, Nagasaki 852-8523, Japan

**Keywords:** NAD^+^, NAMPT, NMN, NR, NMNH, aging/senescence, P7C3, SBI-797812, PNGL, IRW

## Abstract

Aging is a phenomenon underlined by complex molecular and biochemical changes that occur over time. One of the metabolites that is gaining strong research interest is nicotinamide adenine dinucleotide, NAD^+^, whose cellular level has been shown to decrease with age in various tissues of model animals and humans. Administration of NAD^+^ precursors, nicotinamide mononucleotide (NMN) and nicotinamide riboside (NR), to supplement NAD^+^ production through the NAD^+^ salvage pathway has been demonstrated to slow down aging processes in mice. Therefore, NAD^+^ is a critical metabolite now understood to mitigate age-related tissue function decline and prevent age-related diseases in aging animals. In human clinical trials, administration of NAD^+^ precursors to the elderly is being used to address systemic age-associated physiological decline. Among NAD^+^ biosynthesis pathways in mammals, the NAD^+^ salvage pathway is the dominant pathway in most of tissues, and NAMPT is the rate limiting enzyme of this pathway. However, only a few activators of NAMPT, which are supposed to increase NAD^+^, have been developed so far. In this review, we will focus on the importance of NAD^+^ and the possible application of an activator of NAMPT to promote successive aging.

## 1. Introduction

Aging is linked to deterioration due to loss of tissue homeostasis and function that ultimately leads to the occurrence of age-related diseases such as age-related macular degeneration [1], Alzheimer’s disease [2], artherosclerosis [3], immunosenescence [4], as well as cancer [5]. For the past decade, the efforts to understand the genetic and molecular basis of aging have been gaining traction and have led to the proposal of a set of “hallmarks” of aging to facilitate a better conceptualization of the aging process and its underlying mechanisms. These hallmarks include deregulated nutrient sensing, genomic instability, telomere erosion, cellular senescence, epigenetic alterations, mitochondrial dysfunction, stem cell exhaustion, loss of protein homeostasis, and altered cell-to-cell communication [6,7].

Aside from these intrinsic factors, environmental factors also play a crucial role in influencing organismal aging. Diet, in particular, strongly affects aging. For example, several molecules that have been associated with lifespan in experimental model organisms have been confirmed to be functionally involved in cellular nutrient sensing pathways [8,9,10,11,12]. Interestingly, the activity of these molecules—such as Forkhead Box O family (FOXOs), which are involved in regulation of metabolism, stress response and cell proliferation, and death [13], and tuberous sclerosis complex 2 (TSC2), a component of mammalian target of rapamycin complex 1 (mTORC1) inhibitory complex [14], are also found to be under the control of a metabolite master switch called Sirtuin 1, SIRT1 [9]. SIRT1 is a protein deacetylase whose activity is dependent on the availability of a coenzyme called nicotinamide adenine dinucleotide, NAD^+^ [15,16], suggesting that the availability of NAD^+^ is indispensable for counteracting the aging process. Cellular NAD^+^ abundance is predominantly controlled by nicotinamide phosphoribosyltransferase, NAMPT, whose function is responsive to nutrient availability [17,18]. In this review, we highlight the impact of the NAD^+^/NAMPT axis on senescence and aging as well as the potential strategies toward NAMPT activation to counteract senescence and aging.

## 2. NAD^+^ Metabolism

**Figure 1:**
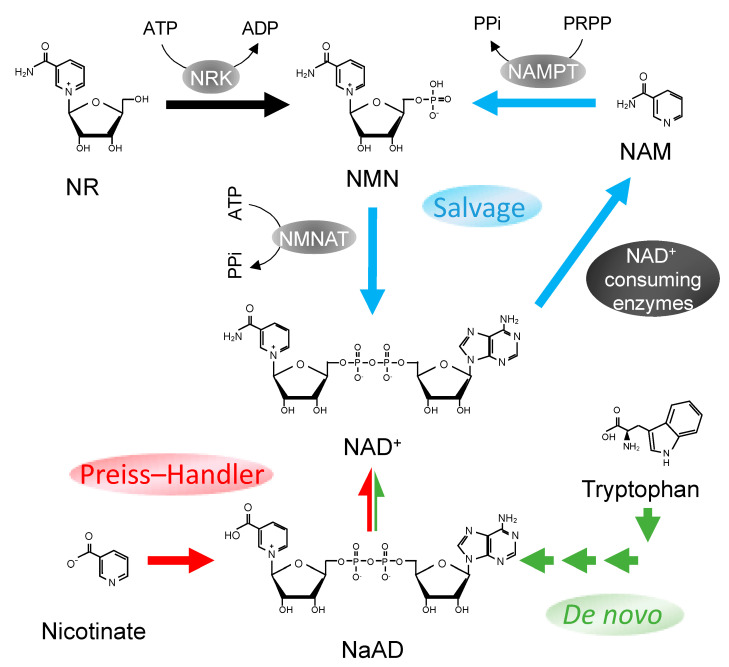
Nicotinamide adenine dinucleotide (NAD^+^) biosynthesis pathways in mammals. Three pathways for NAD^+^ synthesis in mammalian cells: NAD^+^ salvage pathway (blue), Preiss–Handler pathway (red), and de novo pathway (green). NaAD, nicotinic acid adenine dinucleotide; NAM, nicotinamide; NAMPT, nicotinamide phosphoribosyltransferase; NMN, nicotinamide mononucleotide; NMNAT, nicotinamide/nicotinic acid mononucleotide adenylyltransferase, NR, nicotinamide riboside; NRK, nicotinamide riboside kinase; PRPP, 5-phosphoribosyl 1-pyrophosphate; PPi, pyrophosphate.

NAD^+^ consists of two nucleotide molecules that are attached together by their phosphate groups. One of the nucleotides carries an adenine base, while the other, a nicotinamide moiety (Figure 1). NAD^+^ is known to have multiple functions. NAD^+^ is prominently known for its role in redox reactions during energy metabolism. NAD^+^ serves as a hydride shuttle molecule in which H^+^ ion is added to or removed from the nitrogen atom of its nicotinamide moiety, thereby shuttling between its oxidized (NAD^+^) and reduced (NADH) forms. NAD^+^ and NADH also exist in phosphorylated forms (NADP^+^ and NADPH respectively) produced by NAD^+^ kinases and are important in many cellular anabolic reactions.

NAD^+^ is also well known for its role as a cofactor in several processes such as poly (ADP-ribose) polymerization, protein deacetylation, nucleotide phosphorylation, and glycohydrolysis reactions which are carried out by NAD^+^-consuming enzymes such as poly-(ADP-ribose) polymerases (PARPs) [19], sirtuins [15,20], CD73 [21], and CD38 [22], respectively. A new addition to this group of enzymes is sterile alpha and TIR motif-containing 1 (SARM1) [23]. NAD^+^ cleavage activity by SARM1 is carried out by its Toll/interleukin-1 receptor (TIR) domain [24], and this NAD^+^ cleavage activity has been linked to axonal degeneration in response to neuronal injury in mice [23]. Previously thought to be only confined to specific cellular subdomains, NAD^+^ is now known to be excreted by cells and mediates extracellular signaling function. For example, circulating NAD^+^ has been demonstrated to regulate immune response by acting as a pro-inflammatory cytokine [25].

NAD^+^ can be generated via *de novo* NAD^+^ synthesis, where the nicotinamide moiety of NAD^+^ is synthesized from extensive rearrangements of amino acid L-tryptophan through the kynurenine pathway and later coupled to an ADP-ribose group to form NAD^+^ [26] (Figure 1). Another source for NAD^+^ is nicotinic acid (NA), a precursor that is converted to nicotinic acid mononucleotide (NaMN) and subsequently to nicotinic acid adenine dinucleotide phosphate (NaAD) and NAD^+^ through the Preiss–Handler pathway (Figure 1). On the other hand, a rather simpler means of NAD^+^ production is achieved by the synthesis of nicotinamide mononucleotide (NMN) through the NAD^+^ salvage pathway (Figure 1). In this mode of synthesis, NMNs are either produced from nicotinamide riboside (NR) by NR kinase or from nicotinamide (NAM) by NAMPT. NMNs generated are then further adenylated to form NAD^+^ by nicotinamide/nicotinic acid mononucleotide adenylyltransferase (NMNAT). A more detailed description on NAD^+^ syntheses can be found in a review by Yang et al. [27].

However, the reliance on and output from each pathway may vary depending on organism, tissue types, growth condition, and availability of precursors. In prokaryotes such as *Escherichia coli*, *Salmonella typhimurium*, and *Mycobacterium tuberculosis,* NAD^+^ production can be achieved through both *de novo* and Preiss–Handler salvage pathways [28]. Simple eukaryotes such as *Saccharomyces cerevisiae* rely more on the salvage pathway as opposed to the *de novo* pathway, as evident by the comparable NAD^+^ levels recorded between wild type strains and an NAD^+^ *de novo* pathway knockout (*qpt*1∆) yeast strain. This disparity also exists when compared with an NAD^+^ salvage pathway knockout (*npt*1Δ) strain [29]. Meanwhile, in insect species *Drosophila melanogaster* and *Anopheles gambiae,* NAD^+^ *de novo* synthesis is a highly unlikely process due to the absence of an enzyme homologous to mammalian quinolinic acid phosphoribosyltransferase (QPRTase) that links tryptophan catabolism to NAD^+^ synthesis through NaMN [30]. For mammalian cells, NAD^+^ synthesis is achieved predominantly via the NAMPT-mediated NAD^+^ salvage pathway [31] with partial supplementation from the other pathways. Nonetheless, the functional overlap of these mechanisms suggests the importance of NAD^+^ replenishment to cellular physiology across different kingdoms.

## 3. Relationship between Aging/Senescence and NAD^+^

Stemming from the findings that NAD^+^ is a vital factor for the protective role of the Sir2 protein in longevity control in yeast [15] and the remarkable influence Sir2 orthologs have on aging and longevity in worms, flies, as well as humans, Shin-ichiro Imai published a review paper in which he proposed a novel concept of a systemic regulatory network for mammalian aging called “NAD World” [32,33]. The NAD World hypothesis focuses on the central role of NAD^+^ in bridging energy metabolism and aging, a role that is mediated by two key players: the NAMPT enzyme as a “driver” that controls the pace of cellular metabolism through NAD^+^ biosynthesis and SIRT1 as a “mediator” that activates pathways related to survival and aging in response to systemic NAD^+^ availability. The NAD World concept further posits that the communication between the driver and the mediator relies on robust feedback loop signals originating from different tissues and organs, including the hypothalamus, pancreas, adipose tissue, and skeletal muscle [33]. Gradual inability to maintain optimal levels of systemic NAD^+^ may therefore dampen the robustness of the feedback loop signals required to preserve proper metabolic functions [33].

NAD^+^ has indeed been shown to decrease with age in various tissues of mice [34,35,36] and rats [37,38], with a report identifying murine central neurons and pancreatic islets as the fragile points in the maintenance of systemic NAD^+^ homeostasis due to these tissue types having very low intracellular NAMPT levels and having to rely heavily on circulating extracellular NAMPT for NAD^+^ biosynthesis [31,33]. In humans, NAD^+^ levels have been shown to decrease in biopsied aging tissue [39,40]. NAD^+^ content in the extracellular plasma was also found to be altered in association with age [41], while a significant reduction of cellular NAD^+^ was shown in brains of aging but healthy individuals [42] and in human endothelial cells that are involved in age-associated vascular dysfunction [43]. Furthermore, a decreased NAD^+^ level was also observed in cells undergoing senescence in vitro as a function of aging. This was observed in cultured primary human smooth muscle cells, human aortic endothelial cells [44,45], mouse embryonic fibroblast (MEF) cells [46], and rat mesenchymal stem cells (MSC) [47].

The inability to maintain adequate NAD^+^ levels in aging tissue may be attributed to increased activity of NAD^+^ consuming enzymes, such as those that are involved in repairing DNA damage, in cell death and inflammation [39,40,48], as well as in the decline in NAD^+^ synthesis due to downregulation of NAMPT [35,49,50]. Maintaining NAD^+^ level is therefore crucial for tissues, especially to maintain normal physiological function.

## 4. Supplementation of NAD^+^ and Its Precursors Increases NAD^+^ Levels and Ameliorates Signs of Aging

Boosting intracellular NAD^+^ levels has therefore been considered as an option to overcome NAD^+^ depletion and slow down cellular senescence and age-related physiological decline. At the cell level, proofs of concept on administration of exogenous NAD^+^ and its intermediates in culture have yielded promising results. Yang et al. tested the effect of NR supplementation to increase NAD^+^ in several mammalian cell lines—such as human embryonic kidney cell line (HEK293), mouse embryonic stem cell line (AB1), neuroblastoma cell line (Neuro2a)—with all cell lines showing increasing NAD^+^ concentrations ranging from 1.2- to 2.7-fold [17]. NAM supplementation was shown to be able to increase intracellular NAD^+^ level and successfully slowed down senescence of human keratinocytes and human fibroblast cells in vitro [51,52]. Supplementation of NAD^+^ to rat primary neuronal culture reduced DNA damage and prevented cell death [53]. NMN treatment in cerebromicrovascular endothelial cells isolated from aged mice (characterized by loss of capillary-like structures and increased oxidative stress) significantly improved angiogenic processes and attenuated H_2_O_2_ production [54].

Supplementation of NAD^+^ and its intermediates through diet has also showed promising results in the protection against systemic decline of tissue form and function, as evidenced by the increased resistance to age-related pathogenesis and promotion of healthy aging in vivo. In mice, plasma NMN level has been shown to increase as early as 2.5 min after oral NMN administration (300 mg/kg body weight) as these NAD^+^ precursors make their way through the gut lining *en route* to the liver [55]. Plasma NMN level stayed elevated for around 15 min before dropping to normal level, and this was followed by a gradual increase of hepatic NAD^+^ levels 15 to 30 min after administration [55]. A slight NAD^+^ increase was also observed in skeletal muscle and the cortex of the brain 60 min after NMN administration [55]. This demonstrated that supplemented NAD^+^ precursor can immediately perfuse through tissue barriers and rapidly metabolize into NAD^+^ in different major organs.

Pioneering studies have demonstrated that NMN treatment ameliorates glucose intolerance and insulin insensitivity in diet-induced diabetic mice [56,57], and NR treatment protects against diet-induced metabolic abnormalities [58]. A treatment that consists of an NMN dose of 500 mg/kg body weight/day and administered intraperitoneally for 7–10 consecutive days resulted in an increase of NAD^+^ levels in liver, skeletal muscle, and white adipose tissue in high fat diet (HFD)-induced diabetic mice and also rescued impaired insulin tolerance in age-induced diabetic mice while showing no negative effect on non-diabetic cohorts [56,57]. In addition, NMN treatment of similar dosage on mice reared under high fructose diet for 16 weeks that were treated with the same dose of NMN for 16 h prior to analysis was shown to help protect against pancreatic islet inflammation caused by the fructose-rich diet [56,57]. Treatment with the NAD^+^ precursor NR also yielded similar positive outcomes in relation to glucose metabolism. Mice that were fed a high fat diet and supplemented with 400 mg/kg/day NR dose for 10 weeks exhibited a combination of reduced insulin secretion and enhanced glucose, which suggests an overall improvement in insulin sensitivity [58]. Yoshino and colleagues have published an extensive review on the potential therapeutic effects of NMN and NR supplementation [59] and a summary of recent reports on this topic can be found in Table 1. In recent years, supplementation of NAD^+^ or NR in an Alzheimer’s disease mouse model (APP/PS1) was found to increase cortical and hippocampal NAMPT protein levels and to improve cognitive function, inhibiting the accumulation of amyloid beta plaque and body weight gain in these mice [49,60]. Similarly, accumulation of amyloid-like aggregates in muscle tissues, which is an age-related phenomenon found to be conserved across species, and is associated with chronic decline in protein homeostasis, was found to be reduced in aging skeletal muscles of mice after being subjected to NR treatment [61]. On the other hand, NR supplementation in mice showing age-induced non-alcoholic fatty liver disease led to NAD^+^ increases, lower cholesterol and triglyceride levels, and notable improvement in hepatic steatosis and fibrosis [62]. Furthermore, NR or NMN supplementation has helped to alleviate reproductive decline in aging female mice, in which it was found to increase NAD^+^ and NAD(P)H levels in oocytes from mice suffering fertility decline. NAD^+^ precursors-treated mice also showed enhanced fertility and an increase in viable oocytes production, as well as improvements in litter size and the number of living pups [63,64,65]. NMN treatment of aged mice also can bring about similar rejuvenation effects on brain function; in particular, it has been shown to improve spatial working memory function and gait coordination [66], which could be attributed to sirtuin-mediated neurovascular transcriptomic changes [67].

In the past decade, administration of NR as an NAD^+^ booster has garnered attention as a potential effective approach to address age-associated physiological decline in humans. Recent updates include work that showed NAD^+^ precursor NR administration markedly increased NAD^+^ metabolite levels in aged human skeletal muscle [68]. NR has also been tested for its effects on aged persons with notable improvements seen for low density lipoprotein cholesterol level [69] and protection against oxidative stress [70]. In a randomized, double-blind, placebo-controlled, crossover clinical trial, Martens et al. provided further support of the efficacy of chronic NR supplementation in aging adults [71]. In the aforementioned study, a daily dose of 1000 mg of NR supplementation for 6 weeks enhanced NAD^+^ metabolism and lowered the mean value of systolic blood pressure and the appearance of aortic stiffness, both being risk factors for age-related cardiovascular disease.

The latest and exciting findings on NAD^+^ precursor supplementation were reported by Zapata-Perez et al. [72]. The group has identified a reduced form of nicotinamide mononucleotide (NMNH) to have a robust ability to boost NAD^+^ production. NMNH supplementation (500 µM for 24 h) to murine (AML12 and T37i) and human (HepG2, skin fibroblasts, SY5Y, and HeLa) cultured cell lines consistently showed a superior NAD^+^ boosting effect that ranged from 2.5- to 19-fold difference compared with NMN treatment of similar concentration. NMNH treatment (500 µM for 24 h) also was able to increase NAD^+^ levels several fold higher than that of NMN in proximal tubular epithelial cells and was able to confer protection against hypoxia/reoxygenation injury, possibly through restoring disrupted electron transfer chain flux and enhancing mitochondrial function [72].

The effect of NMNH supplementation in vivo on NAD^+^ levels was also explored by introducing 250 mg/kg of NMN or NMNH into C57BL/6N mice through intraperitoneal injections [72]. Rapid increase of blood NAD^+^ levels was recorded for both NMN and NMNH, with NMNH showing a higher fold increase than that of NMN after 60 min of administration. More strikingly, while NMN only managed to increase blood NAD^+^ levels for 4 h before returning to basal level, NMNH was found to be able to sustain a 2-fold higher level of NAD^+^ for at least 20 h [72]. The ability to stimulate higher biosynthesis and prolonged availability of NAD^+^ demonstrated NMNH superiority as an NAD^+^ enhancer and would potentially lead to new avenues for therapeutic applications in human aging.

It is interesting to note the NAD^+^ boosting effect of these supplementations might not be as straight forward as initially thought due to the recent discovery that gut microbiota may also play a role in the NAD^+^ boosting effect of dietary NAM. Shats and colleagues recently reported that nicotinamidase (PncA) produced by microbes in the gut actively converts NAM to NA [73]. NA is then taken up by colonic epithelial cells and further converted to NAD^+^. This essentially reroutes the NAM from NAD^+^ salvage pathway to the Preiss–Handler pathway. Furthermore, by tracing the fate of isotope-labeled NAM after treatments, NAD^+^ molecules produced through this mode of NAD^+^ biosynthesis have also been found to represent the major portion of the elevated NAD^+^ pool in murine hepatic cells [73]. This demonstrated that a host–microbe metabolic interaction can influence the efficacy of certain NAD^+^ boosting nutraceuticals. It is known that gut microbiome composition can differ between aging individuals that are of different health status [74]. Therefore, information on the gerobiotic status (composition of probiotic and parabiotic strains that are able to impact fundamental mechanisms of aging; reviewed by Tsai et al. [75]) of aging individuals undergoing NAD^+^ supplementation should be monitored through the course of treatment to ensure that accurate conclusions of the treatment outcome are made.

## 5. NAMPT Overexpression Shows Resistance against Senescence

Genetic manipulation of the NAD^+^ synthesis pathways provides another avenue to improve cellular NAD^+^ availability. One popular target for manipulation is NAMPT, the rate-limiting enzyme of the NAD^+^ salvage pathway (Figure 1) [76]. Consistent with NAD^+^ depletion with aging, NAMPT levels also have been reported to decline in aged tissues of rats [77] and mice [35,49,78]. It is worthy to note that as NAD^+^ is produced from NMN by NMNAT, targeting NMNAT is a tractable means by which to boost NAD^+^ production. However, overexpression of *Nampt*, but not *Nmnat,* was able to increase NAD^+^ level in mouse fibroblast [76]. This indicates that NAMPT serves as the rate-limiting enzyme for this cycle and a promising target for NAD^+^ synthesis enhancement.

*Nampt* knockdown in middle-aged mice reduces hepatic NAD^+^ to levels found in aged mice and causes hepatic tissue inflammation [35]. In contrast, *Nampt* overexpression can improve murine endothelial progenitor cell proliferation by preventing the onset of senescence [79], while *Nampt* overexpression attenuates cell senescence in both aged rat MSCs [38] and MSCs undergoing serial expansion in vitro [47]. Overexpression of the *Nampt* gene has been previously reported to increase intracellular NAD^+^ level in skeletal muscle of mice [80] and to confer protection against cell death in murine cardiac muscles [81] and neuronal cells [82]. In human cells, *Nampt* overexpression also increases intracellular NAD^+^ levels and extends replicative lifespan in human aortic endothelial cells [45] and human smooth muscle cells [44].

Our group has also generated several lines of transgenic mice overexpressing the human *Nampt* gene (*Nampt*-Tg) and has reported that intracellular NAD^+^ levels in MEF derived from these transgenic lines were increased approximately 2-fold [46]. We observed that these *Nampt*-Tg MEF cells have higher resistance to replicative senescence and are able to proliferate more times as compared with wild type MEF cells. This protective effect is most likely brought about by increased expression of antioxidant genes, such as *sod2* and *catalase*, via SIRT1 activation. SIRT1 activity in these *Nampt*-Tg MEF cells was found to be increased, which is expected since SIRT1 activity is dependent on NAD^+^ availability. Furthermore, these *Nampt*-Tg MEF cells have also been shown to be resistant to cellular senescence induced by oxidative and endoplasmic reticulum (ER) stress [83]. In particular, *Nampt*-Tg MEF cells subjected to tunicamycin treatment showed enhanced *Xbp1* splicing and subsequently higher upregulation of several downstream target genes involved in the unfolded protein response (UPR) signaling pathways when compared with similarly treated wild type MEFs. Thus, our reports indicate that the NAMPT/NAD^+^ axis protects against cellular senescence, irrespective of the type of senescence, suggesting that NAMPT activation instead of direct NAD^+^ enhancement is the alternative strategy to delay or ameliorate the aging/senescence process.

## 6. Possibility of NAMPT Activation by Small Molecules

Exercise and nutrient restriction can increase NAMPT in mammals [17,77]. NAMPT levels fluctuate to match cellular NAD^+^ demand, especially during physical exercise and nutritional perturbation such as calorie restriction and diet variation [56,84,85]. Maintenance of NAD^+^ homeostasis is also influenced by the circadian clock through its control on *Nampt* gene expression [86,87,88]. Exercise is also consistent in enhancing NAMPT levels in human muscles [89,90]. However, exercise may not be suitable for certain people with difficulty in performing physical activities. In addition, individuals with specific dietary needs may not be amenable to such nutrient restriction measures such as fasting. Screening and the development of compounds that can serve as direct NAMPT activators may therefore be critical for certain individuals. This field of research is actively being explored, considering the enormous potential application of NAMPT activators for therapy, several potential candidates of which have been identified so far.

P7C3, which is a type of aminopropyl carbazole chemical having proneurogenic and neuroprotective properties in both newborn and adult mice [91], has been reported as the first activator of NAMPT (Table 2) [92,93]. P7C3 can target human recombinant NAMPT and enhance NMN production in a dose-dependent manner, and treatment with P7C3 was able to rescue cultured human cells from NAD^+^ depletion due to doxorubicin treatment [92]. P7C3 also has been demonstrated to increase the NAD^+^ level in mouse brain [94]. The mechanism of action of P7C3 is still unclear in terms of its specific binding site to NAMPT. In addition, whether P73C directly activates NAMPT or rather it exerts its effect by blocking endogenous NAMPT inhibitors through competitive binding to NAMPT is not clear. P7C3 has been shown to possess neuroprotective activity for hippocampal neurogenesis [95] as well as to slow down chronic neurodegeneration and to restore cognitive function in the context of traumatic brain injury in mice [93,96]. In addition, P7C3 has also been shown to promote adult neurogenesis in the hippocampus of a non-human primate model [97]. This is particularly encouraging as neurogenesis declines with age in the adult hippocampus of mammals [98]. Nevertheless, the role of P7C3 in boosting NAMPT activity is still in dispute [99]. It therefore remains to be seen if P7C3 would activate NAMPT in different tissue types and whether or not P7C3 is beneficial against the aging process and in broader age-associated decline in tissue function.

SBI-797812 is another chemically synthesized compound that has been reported to possess the ability to activate human recombinant NAMPT [99]. SBI-797812, which is structurally similar to active-site directed NAMPT inhibitors, was identified by high-throughput screening of a small molecule library using a protein thermal shift assay [99]. This compound was found to enhance ATPase activity of NAMPT and stabilizes phosphorylated NAMPT, leading to a boost in NMN synthesis. Using 13C/15N-labelled NAM tracing, the effect of SBI-797812 treatment was shown to induce a 5-fold increase in cellular NAD^+^ levels. SBI-797812 treatment led to increased NMN and NAD^+^ levels in human alveolar basal epithelial adenocarcinoma (A549) cells and was also found to inhibit NAD^+^ mediated feedback inhibition on NAMPT.

Notoginseng leaf triterpenes (PNGL), which are the total saponins extracted from *Panax* notoginseng stems and leaves [100,101], are novel natural compounds that have recently been shown to activate *Nampt* gene expression [102]. PNGL has already been shown to possess antioxidative effects against a human neuroblastoma cell line [101,103]. Furthermore, using rats with cerebral artery occlusion/reperfusion, PNGL has been shown to decrease infarct volume and brain water content, improve neurological functions, and alleviate blood–brain barrier disruption, resulting in protection from neuronal loss [104]. Xie and colleagues have recently reported that the neuroprotective effects of PNGL against brain ischemia and reperfusion in rats were due to an increase in NAMPT protein levels and sustaining NAD^+^ levels in the cortex and hippocampus along with activation of the SIRT/MnSOD/PGC-1α axis [105]. Although it remains to be seen exactly how PNGL would directly or indirectly activate *Nampt* gene expression, PNGL might turn out to be a potent NAD^+^-boosting compound in vivo.

IRW (Ile-Arg-Trp) is a natural peptide-based small molecule found to have NAMPT-activating effects [106]. This bioactive tripeptide is prepared from egg white protein ovotransferrin through enzymatic digestion [107] and has been reported to possess an inhibitory effect on angiotensin-converting enzyme (ACE) [107] and a potential anti-inflammatory effect [108]. IRW treatment on cultures of rat skeletal muscle (L6) cells has resulted in an increase of intracellular NAMPT protein abundance by 2.2-fold [106]. NAMPT protein levels were also found to be increased in both muscle and liver tissues (an average of 4- and 6-fold, respectively) of C57BL/6J HFD mice after completing an IRW treatment regime (45 mg/kg body weight dosage; mice were fed HFD for six weeks followed by HFD + IRW for another eight weeks) [106]. The increase in NAMPT abundance was accompanied by an increase in NAD^+^ levels in both aforementioned tissue types [106]. IRW most likely exerted its modulatory influence on NAMPT activity at the gene transcription level, based on the finding that transcript levels of *Nampt* in both muscle and liver tissues were increased upon IRW treatment [106].

## 7. Closing Remarks

Declining cellular NAD^+^ levels has become central to explaining many age-related pathophysiologies. As such, measures to slow down the decline of NAD^+^ as well as to boost the production of NAD^+^ in aging tissues using chemical and genetic approaches are actively being explored. In contrast to the growing data showing the positive effects of NAD^+^ precursors on aging/senescence in rodents and also humans, studies of small compounds that activate NAMPT are still in their infancy. Nonetheless, it is exciting to imagine that combined administration of NAD^+^ precursors and small compounds activating NAMPT may be an attractive and powerful strategy to effectively prevent and cure age-related diseases in the future. Further studies, especially on the exploration and development of NAMPT activators, are therefore needed to help expand our knowledge of the underlying mechanism of aging, and to work toward achieving healthy and productive aging.

## Figures and Tables

**Table 1:** Effects of administration of NAD^+^ or precursors in vivo.

Compound	Dose	Duration	Outcome	Ref.
NAD^+^	30 mg/kg, IP *	4 weeks	alleviated spatial learning and memory, and reduced senile plaques in Alzheimer’s disease model mouse	[49]
NR	400–460 mg/kg body weight, food	3 months	inhibited accumulation of Ab in Alzheimer’s disease model mouse	[60]
NR	400 mg/kg body weight, food	8 weeks	increased muscle homeostasis and attenuated amyloid accumulation in mouse muscle	[61]
NR	400 mg/kg body weight, food	3 months	protective effect against aging-induced NAFLD-like hepatic dysfunction	[62]
NR	400 mg/kg body weight, drinking water	4 months	restored oocyte quality and fertility in aged mice	[63]
NMN	0.5–2 g/L, drinking water	4 weeks	restored oocyte quality and fertility via SIRT2 activation in aged mice	[64]
NMN	200 mg/kg body weight, IP **	10 days	restored oocyte quality and fertility in aged mice	[65]
NMN	500 mg/kg body weight, IP **	2 weeks	rescued neurovascular coupling responses and improved spatial working memory function and gait coordination	[66]
NR	1 g/day, OA	3 weeks	increased in NAD^+^ metabolome in aged human skeletal muscle	[68]
NR	250 or 500 mg/day, OA	8 weeks	increased in NAD^+^ levels in whole blood and improvement on low density lipoprotein cholesterol level in healthy older subjects	[69]
NR	250 mg, OA	2 h	decreased oxidative stress in healthy old subjects	[70]
NR	2 × 250 mg /day, OA	6 weeks	increased in NAD^+^ levels in PBMCs of healthy middle-aged and older subjects	[71]
NMNH	250 mg/kg body weight, IP	<24 h	increased in NAD^+^ levels in blood and several organs of mouse in folds higher than NMN	[72]

IP, intraperitoneal; *, injection every other day; **, injection every day; OA, orally administration.

**Table 2:** Effects of compounds on NAMPT in vitro/in vivo.

Compound	Dose	Duration	Outcome	Ref.
P7C3	0.3–3 mM, in vitro		Increase in NAMPT activity and NAD^+^ in vitro	[92]
P7C3	30 mg/kg body weight, IP **	2 h	increase in NAD^+^ in mouse brain	[94]
SBI-797812	0.4–10 mM in vitro 20 mg/kg body weight, IP *	4 h 4 h	Increase in NAMPT activity and NAD^+^ in vitro and in mouse liver	[99]
PNGL	6.25 mg/ml, in vitro		Increase in NAMPT expression and NAD^+^ and amelioration of neuronal injury in vitro	[102]
PNGL	73–292 mg/kg body weight, IA	2 weeks	Increase in NAMPT expression and NAD^+^ and amelioration of mitochondrial oxidative injury in rat brain	[105]
IRW	50 mM in vitro 45 mg/kg body weight, OA	24 h 8 weeks	Increase in NAMPT expression and NAD^+^ in vitro and in mouse liver and muscle tissues	[106]

IP, intraperitoneal; *, injection every other day; **, injection every day; IA, intragastric administration; OA, orally administration.

## Data Availability

Not applicable.
